# Reading Level and Suitability of Congestive Heart Failure (CHF) Education in a Mobile App (CHF Info App): Descriptive Design Study

**DOI:** 10.2196/12134

**Published:** 2019-04-25

**Authors:** Ponrathi Athilingam, Bradlee Jenkins, Barbara A Redding

**Affiliations:** 1 College of Nursing University of South Florida Tampa, FL United States

**Keywords:** health literacy, reading level, patient education, heart failure, mobile app

## Abstract

**Background:**

Education at the time of diagnosis or at discharge after an index illness is a vital component of improving outcomes in congestive heart failure (CHF). About 90 million Americans have limited health literacy and have a readability level at or below a 5th-grade level, which could affect their understanding of education provided at the time of diagnosis or discharge from hospital.

**Objective:**

The aim of this paper was to assess the suitability and readability level of a mobile phone app, the CHF Info App.

**Methods:**

A descriptive design was used to assess the reading level and suitability of patient educational materials included in the CHF Info App. The suitability assessment of patient educational materials included in the CHF Info App was independently assessed by two of the authors using the 26-item Suitability Assessment of Materials (SAM) tool. The reading grade level for each of the 10 CHF educational modules included in the CHF Info App was assessed using the comprehensive online Text Readability Consensus Calculator based on the seven most-common readability formulas: the Flesch Reading Ease Formula, the Gunning Fog Index, the Flesch-Kincaid Grade Level Formula, the Coleman-Liau Index, the Simplified Measure of Gobbledygook Index, the Automated Readability Index, and the Linsear Write Formula. The reading level included the text-scale score, the ease-of-reading score, and the corresponding grade level.

**Results:**

The educational materials included in the CHF Info App ranged from a 5th-grade to an 8th-grade reading level, with a mean of a 6th-grade level, which is recommended by the American Medical Association. The SAM tool result demonstrated adequate-to-superior levels in all four components assessed, including content, appearance, visuals, and layout and design, with a total score of 77%, indicating superior suitability.

**Conclusions:**

The authors conclude that the CHF Info App will be suitable and meet the recommended health literacy level for American adult learners. Further testing of the CHF Info App in a longitudinal study is warranted to determine improvement in CHF knowledge.

## Introduction

### Heart Failure Education

Education is key to improve knowledge and persuade patients with congestive heart failure (CHF) to practice recommended daily self-management tasks of weight and symptom assessment, diet, exercise, and pharmacological therapy [1]. Evidence indicates that traditional patient education using printed materials does not support self-management skill development [1]. New patient-teaching strategies are needed for prolonged engagement of patients to support the development of tactical and situational skills [2]. The incidence of CHF is strongly dependent on age, with an estimated incidence of 1% at age 65 that approximately doubles with each decade thereafter, affecting 6.5 million Americans [3]. According to the Centers for Medicare and Medicaid Services, nearly 20% of all Medicare patients are readmitted to the hospital within 30 days and 34% are readmitted within 90 days of discharge [4]. As many as 79% of readmissions are considered preventable. The prospective EuroHeart Failure Survey (N=2331) from 24 countries followed patients with CHF for 12 weeks and reported that about half of the patients (49%) recalled receiving advice to weigh themselves at discharge [5]. American health care puts a great deal of emphasis on patient autonomy and patients' “right to know.” The American College of Cardiology/American Heart Association clinical guidelines include written educational instruction at the time of discharge as one of the core performance measures to improve CHF outcomes [6]. However, adequate communication skills and health literacy level is important for patients to understand the discharge instructions given, so they can follow them appropriately at home [7]. Inadequate communication skills may not mean resistance to the treatment plan or poor intellect, but rather a low literacy level. Thus, assessing and addressing health literacy levels of patients and reading levels of patient educational materials are warranted for patient safety and to improve outcomes among the elderly.

In recent years, patient-centered mobile health technologies have emerged as a way to actively engage patients in their health care decision-making process. Patients who are engaged as decision makers in their care tend to be healthier and have better outcomes. This is supported by the national survey of 1604 mobile phone users, where 76% of mobile phone users were constantly connected by technology [8]. African Americans and Latinos are reported to be 50% more dependent on mobile phones compared to the white population; about 80% of adults over the age of 65 use mobile phones and 42% are advanced-feature mobile phones [9]. Older adults aged 65 and over with no experience in technology are reportedly using mobile phones to manage daily self-management of chronic diseases [10,11]. Thus, embedding health education in a mobile platform is proposed to improve patient engagement, facilitate communication, help overcome health challenges, and improve disease management. However, health literacy could affect people's ability to navigate the health care system, including filling out complex health information forms and engaging in both self-care and chronic disease management [12]. This paper addresses the suitability and the reading-level assessment of patient educational materials included in the mobile app *CHF Info App*.

### Health Literacy

Health literacy is the ability to understand health information and to use that information to make good decisions about one’s health and health care [13]. According to the US Department of Health and Human Services, over a third of US adults are reported to have difficulty with common health tasks, such as following directions on a prescription drug label, adhering to a childhood immunization schedule, or understanding the direction of a medication schedule from the instructions found in the medication container [13]. Health literacy also encompasses the educational, social, and cultural factors that influence the expectations and preferences of the individual [14]. According to the Centers for Medicare and Medicaid Services, about 70% of adults older than age 60 had difficulty using print materials and 80% had difficulty using documents such as forms or charts [15]. Limited health literacy affects adults in all racial and ethnic groups. The proportion of adults with basic or below basic health literacy ranges from 28% of white adults to 65% of Hispanic adults [13]. Approximately 90 million Americans have limited health literacy and have a readability level at or below the 5th-grade level [16]. Although half of adults without a high school education may have below basic health literacy skills, even high school and college graduates can also have limited health literacy [14]. The Joint Commission recommended that in order to self-manage their own health care, individuals must be able to locate health information, evaluate that information for relevance and credibility, and analyze risks and benefits. For those with limited literacy skills, self-management may be too much of a challenge to be overcome, especially if such challenges are undiscovered or ignored [7]. Suitability of educational materials (ie, ease of understanding and acceptance) and readability (ie, reading difficulty) at the level of patient education or understanding have been suggested as strategies to improve the knowledge of the CHF patient. Despite the American Medical Association (AMA) recommendation to provide all patient educational materials at a 6th-grade reading level, many educational materials do not abide by the rule [17].

With advances in medical science, health educational information can overwhelm people, even those with advanced literacy skills. Patient educational materials are potentially effective at improving patient comprehension and influencing health behaviors, especially if they are written at appropriate reading levels for patients. Currently, most institutions include links for patient education within electronic health records (EHRs). An assessment of reading levels of three commonly used patient education links in an EHR study reported varied reading levels: 11th-13th grade for EBSCO, 14th-17th grade for MedlinePlus, and 11th grade for Micromedex—grade levels above the 12th grade refer to the college or university level and beyond [18]. Similarly, an assessment of 339 online ophthalmic patient educational materials demonstrated a varied reading level ranging from the 10th to the 17th grade [19], which is higher than the AMA-recommended 8th-grade reading level. However, no reading levels are assessed on patient educational materials included in mobile apps. Recent reviews evaluated the quality of mobile apps regarding the ease of use, reliability, quality, scope of information, and aesthetics using the Mobile App Rating Scale, but not regarding the reading level of patient educational materials [20,21].

Therefore, the original theory-based development of the CHF Info App with 10 education modules was assessed for reading levels to meet those recommended by the AMA. The modules included in the app were based on educational materials from the Heart Failure Society of America (HFSA) with their approval; a link to detailed HFSA material is provided in the app for patients to navigate [22]. The CHF Info App was developed by educational technology and computer engineering students from the University of South Florida. The app was beta-tested on a small sample of patients and health care providers for usability [22] by the educational technology students. The content included was approved by cardiologists during beta-testing [22]. The educational material included in the CHF Info App evolved and additions were made based on input from patients and providers after beta-testing [22]. Given the influence of health literacy on understanding educational material, assessing for suitability and reading level of patient educational material was deemed a necessary next step before making the CHF Info App available for patient use. Therefore, the authors sought to determine the suitability and readability level of the materials included in the CHF Info App to meet the requirements of the AMA recommendations.

## Methods

A descriptive design was used to assess the suitability and readability of the patient educational materials included in the CHF Info App. The Suitability Assessment of Materials (SAM) tool is composed of a 26-item Likert scale with four categories including message content, text appearance, visuals, and layout and design. The SAM instrument is a systematic tool that assesses printed health-related educational resources in a short amount of time. The validated SAM tool was used to assess the CHF Info App [23]. Two of the authors (PA and BR) independently assessed the suitability of the CHF Info App using the SAM instrument. These authors, both with doctoral-level education, objectively followed the SAM tool and coded each of the 10 modules according to content, appearance, visuals, and layout and design. Each item was scored as *superior* (2 points), *adequate* (1 point), or *not suitable* (0 points). The given score was divided by the total possible score to obtain a percentage; a score of 0%-39% is considered *not suitable*, 40%-69% is considered *adequate*, and 70%-100% is considered *superior*. Conflict between the coders was resolved by consensus and a workflow for improvement of the app was developed.

There are several predominant tools available online to measure the reading level of patient educational materials. The authors used the Text Readability Consensus Calculator, an online readability consensus assessment formula, which calculates reading level based on the seven most-common reading formulas to calculate the average reading grade level, reading age, and text difficulty of the text [24]. The prominent measures of readability documented in the literature are the Flesch Reading Ease Formula, which indicates that the best text should contain shorter sentences and words. A score between 60 and 70 is largely considered acceptable [25]. The Flesch-Kincaid Grade Level Formula presents a score as a US grade level, making it easier for teachers, parents, librarians, and others to judge the readability level of various books and texts [26]. The Simplified Measure of Gobbledygook Readability Formula estimates the years of education needed to understand a piece of writing, particularly for checking health messages [27]. The Gunning Fog Index estimates the years of formal education a person needs to understand a piece of text on the first reading. For instance, a Gunning Fog Index of 12 requires the reading level of a US high school senior [28]. The Fry Formula is calculated as the average number of sentences (y-axis) and syllables (x-axis) per 100 words. These averages are plotted onto a specific graph; the intersection of the average number of sentences and the average number of syllables determines the reading level of the content [29]. The Dale-Chall Formula is unique because, unlike other formulas that use word length to assess word difficulty, the Dale-Chall Formula uses sentence length and counts of “hard” words to calculate the US grade level [30]. In addition, the Linsear Write Formula is included to calculate the readability of technical manuals; also, the Automated Readability Index was designed to gauge the understandability of a text that produces an approximate representation of the US grade level needed to comprehend the text [24]. Therefore, the 10 education modules of the CHF Info App were assessed using the comprehensive Text Readability Consensus Calculator Formula to make sure the CHF Info App meets the AMA recommendations.

## Results

A suitability assessment of patient educational materials from the CHF Info App was performed by two of the authors using the SAM tool. Both of the authors (PA and BR) with doctoral-level education were qualified to assess the quality of the material objectively using the SAM tool. Each item was scored as *superior* (2 points), *adequate* (1 point), or *not suitable* (0 points), as mentioned above. The result of the assessment demonstrated *adequate*-to-*superior* levels of agreement in all four components: content, appearance, visuals, and layout and design. The only item found to be inadequate or that was not specifically addressed in the CHF Info App was cultural difference. The authors agreed that the educational material provided in the CHF Info App was general and culturally neutral and did not address any one cultural preference. The CHF Info App was assessed as *superior* with a score of 77%. See Table 1 for detailed results.

The reading grade level for each of the 10 CHF educational modules included in the CHF Info App was assessed using the comprehensive online Text Readability Consensus Calculator formulas [24]. The reading level included the text-scale score, the ease-of-reading score, and the corresponding grade level. The materials included in the CHF Info App ranged from 5th-grade to 8th-grade reading levels with an average of a 6th-grade level, which meets the AMA recommendation (see Table 2).

**Table 1:** Suitability Assessment of Materials for patient education of the CHF^a^ Info App.

Appraisal of components and related questions		Assessment based on score		
		Inadequate	Adequate	Superior
**Appraisal of message content**				
	Does the material explain the purpose and benefits from the patient’s view?		X	
	Is the content limited to a few essential main points that the majority of the target population will benefit from?			X
	Are behaviors and skills emphasized rather than just facts?			X
	Are readers provided with opportunities for small successes?		X	
	Are key points reviewed at the end of each section or page?		X	
	Is the material sensitive to cultural differences?	N/A^b^	N/A	N/A
	Is the new information placed in the context of the patients’ lives?		X	
	Are readers told what they should get from the material and what they can do to improve their health?			X
	Is the organization of the paragraphs and sentences conducive to easy reading?			X
	Are instructions broken down into easy-to-read parts?			X
	Is the material interactive? Does it encourage the patient to write, answer questions, ask questions, cut out forms, etc?			X
**Appraisal of text appearance criteria**				
	Is the font size no smaller than 12pt-14pt? Is zoom function available?			X
	Is easy-to-read font used? Are there no fancy scripts or lettering?			X
	Are bold and underline used instead of ALL CAPS and italics?			X
	Are colors used to promote easy reading (ie, dark fonts on light backgrounds are best)?			X
	Are overall sharp contrast and large font used?			X
**Appraisal of visuals**				
	Do the visuals all help communicate your messages in a literal manner (ie, no abstract symbols)?		X	
	Are the visuals culturally relevant and sensitive?		X	
	Are the visuals easy for your readers to follow and understand? For example, if showing a sequence, are the steps numbered and labeled?		X	
	Are internal body parts or small objects shown in context and in a realistic manner?		X	
	Are the visuals professional and appropriate for an adult audience?			X
	Are the visuals free of distracting details that take away from the main idea?			X
	Do all of the graphics contribute to your message?		X	
	Are examples given for any lists, charts, or diaries that readers are supposed to complete?		X	
**Appraisal of layout and design**				
	Is the cover effectively designed?		X	
	Are messages organized so they are easy to act on and recall (headings, subheadings, *chunking*, etc)?			X
	Is there a lot of white space? Is there no dense text?		X	
	Is the text easy for the eye to follow? For example, bullets, paragraph shape (40-50 characters wide is best), and text boxes.			X

^a^CHF: congestive heart failure.

^b^N/A: not applicable.

**Table 2:** Reading-level assessment of the CHF^a^ Info App.

Scoring formula	Text-scale score	Ease-of-reading score	Grade level
Flesch Reading Ease Formula	84.8	Easy	N/A^b^
Gunning Fog Index	6.8	Fairly easy	N/A
Flesch-Kincaid Grade Level Formula	4.7	Easy	5th grade
Coleman-Liau Index	8.0	Fairly easy	8th grade
Simplified Measure of Gobbledygook Index	4.4	Easy	4th grade
Automated Readability Index	5.9	Easy	5th and 6th grade
Linsear Write Formula	6.1	Easy	6th grade

^a^CHF: congestive heart failure.

^b^N/A: not applicable.

**Table 3:** Comparison of words and text of standard US high school and adult readers and the CHF^a^ Info App.

Measure	Average for US high school and adult readers	Average in the CHF Info App
Sentence length (number of words), mean (SD)	13-16 (1.3)	13 (0.7)
Reading level	7th and 8th grade	6th grade
Three-syllable text, %	12-14	4

^a^CHF: congestive heart failure.

In addition to the reading level, the online Text Readability Consensus Calculator provided a comparison of words and syllables, sentence length, and texts included in the CHF Info App corresponding with the standard of US high school and adult readers. The results showed appropriate sentence length, reading level at the 6th grade, and an average three-syllable text of 4%; the results also meet the AMA recommendation (see Table 3).

## Discussion

### Principal Findings

The findings of the assessment of the patient educational material included in the CHF Info App indicated a reading level of 5th-8th grade, with a mean of a 6th-grade level. This result is consistent with the recommendation from the AMA [17]. The ability to obtain and understand basic information about health in order to make informed decisions is vital and contributes to the complex area of health literacy. Although the use of medical terminology in patient educational material is often unavoidable, it has a profound impact on readability because of the use of polysyllabic medical terms, and the CHF Info App included fewer polysyllabic terms. The CHF Info App was made up of 4% of three-syllable text, which is much lower than the recommended average three-syllable text of 12%-14%; in addition, the average sentence length of 13 words met the standard for US high school and adult learners. A study by Chen et al showed that health literacy is associated with CHF knowledge, longitudinally (*P*<.001), among 51 patients with CHF with a mean age of 65 years [31]. Therefore, the authors conclude that the CHF Info App will be suitable and meet the health literacy level recommended for adult learners. Low health literacy was consistently associated with more hospitalizations; decreased ability to demonstrate taking medications appropriately; decreased ability to interpret labels and health messages; and, among elderly persons, poorer overall health status and higher mortality rates. Health literacy was independently associated with knowledge (*P*<.001), however, it was not related to self-care [32]. Therefore, our next step is to test the CHF Info App in a longitudinal study to measure improvement in CHF knowledge and self-care.

The reading-level assessment was complemented by the use of the SAM instrument, which assessed the content, appearance, visuals, and layout and design of the materials. The components were assessed as *adequate* to *superior* for all 26 items on the SAM instrument, with a total score of 77% indicating *superior*. Except for cultural difference, the other items were found to be *adequate* or *superior*. The authors concluded that the contents included in the CHF Info App are general, using common words, and are culturally neutral. However, further testing is needed among a diverse population to determine its usability and potential efficacy, since poor health literacy partially explains racial disparities in some outcomes [33]. Evidence supports the use of mobile phone-based telemonitoring and educational support for patients with CHF [11].

### Limitations

One limitation is that this was a descriptive study that assessed the readability and suitability of a mobile phone-based CHF education app—the CHF Info App. The other major limitation is that the CHF Info App is available only in the English language. Further testing on a larger sample including elderly persons and a longitudinal follow-up are warranted to determine if the patient educational material included in the CHF Info App will improve CHF knowledge and self-care. Although the readability measures used in this study do address reading grade level and ease of reading, through assessment of word difficulty and sentence length, they do not consider other factors that may affect comprehension of health educational materials, such as cultural appropriateness, learning stimulation, and motivation. Although the suitability of educational material includes many factors, reading grade level is foundational to any patient educational material.

### Clinical Implications

All patient educational material, whether in paper form or an app version, should identify reading level and how it was measured to provide further guidance for patients and health care providers to make sure that they meet the AMA recommendations. Future research should focus on continued assessment of health educational materials used for diverse populations and settings and investigation of readability measures among CHF patients.

## Abbreviations

AMA: American Medical Association
CHF: congestive heart failure
EHR: electronic health record
HFSA: Heart Failure Society of America
SAM: Suitability Assessment of Materials
